# Survival Rate of 1008 Short Dental Implants with 21 Months of Average Follow-Up: A Retrospective Study

**DOI:** 10.3390/jcm9123943

**Published:** 2020-12-05

**Authors:** João Caramês, Ana Catarina Pinto, Gonçalo Caramês, Helena Francisco, Joana Fialho, Duarte Marques

**Affiliations:** 1 Faculty of Dental Medicine, University of Lisbon, 1600-277 Lisbon, Portugal; carames@campus.ul.pt (J.C.); helenafrancisco@campus.ul.pt (H.F.); 2 Instituto de Implantologia, 1070-064 Lisbon, Portugal; anacatarina.pinto@institutoimplantologia.com (A.C.P.); caramesgoncalo@gmail.com (G.C.); 3 LIBPhys-FCT UID/FIS/04559/2013, Faculty of Dental Medicine, University of Lisbon, 1600-277 Lisbon, Portugal; 4 Escola Superior de Tecnologia e Gestão de Viseu, Centro de Estudos em Educação, Tecnologias e Saúde, 3504-510 Viseu, Portugal; jotafialho@hotmail.com

**Keywords:** short implant, implant length, survival rate, prognosis

## Abstract

This retrospective study evaluated the survival rate of short, sandblasted acid-etched surfaced implants with 6 and 8 mm lengths with at least 120 days of follow-up. Data concerning patient, implant and surgery characteristics were retrieved from clinical records. Sandblasted and acid-etched (SLA)-surfaced tissue-level 6 mm (TL6) or 8 mm (TL8) implants or bone-level tapered 8 mm (BLT8) implants were used. Absolute and relative frequency distributions were calculated for qualitative variables and mean values and standard deviations for quantitative variables. A Cox regression model was performed to verify whether type, length and/or width influence the implant survival. The cumulative implant survival rate was assessed by time-to-event analyses (Kaplan–Meier estimator). In all, 513 patients with a mean age of 58.00 ± 12.44 years received 1008 dental implants with a mean follow-up of 21.57 ± 10.77 months. Most implants (78.17%) presented a 4.1 mm diameter, and the most frequent indication was a partially edentulous arch (44.15%). The most frequent locations were the posterior mandible (53.97%) and the posterior maxilla (31.55%). No significant differences were found in survival rates between groups of type, length and width of implant with the cumulative rate being 97.7% ± 0.5%. Within the limitations of this study, the evaluated short implants are a predictable option with high survival rates during the follow-up without statistical differences between the appraised types, lengths and widths.

## 1. Introduction

Implant therapy for the rehabilitation of partially or totally edentulous patients has become increasingly widespread in the last decades. In 1982, Branemark [1] first described the use of dental implants with prosthetic survival rates of 81% and 100% at 15 years for the maxilla and the mandible, respectively. Since then, implants have been usually considered as a treatment option for edentulous patients.

The bone availability for implant placement is limited by anatomical structures such as the maxillary sinus and the inferior alveolar nerve [2,3,4,5]. Thus, nowadays, one of the greatest challenges is placing an implant in the atrophic maxillae due to limited ridge height, which in turn implies a higher risk of damaging important anatomical structures.

A wide range of surgical procedures have been proposed to overcome these dimensional limitations in both the atrophic maxilla and the mandible, including sinus floor augmentation, tilted or zygomatic implants, bone block grafts for onlay or inlay/interpositional grafts, distraction osteogenesis, guided bone regeneration (for alveolar defects, socket grafts, lateral and vertical augmentations) and inferior alveolar nerve transposition [5,6,7,8,9,10,11,12,13,14,15,16]. Despite the high success rate of some of these techniques, such as bone grafting, they are associated with constraints such as the need for an appropriate healing time, which implies increased treatment time and cost, increased postoperative morbidity and risk of complications since they are operator-sensitive techniques where the surgeon’s skills and experience are key factors for success [10,17,18]. Moreover, except for the sinus floor augmentation, data on the predictability of those procedures are scarce [9]. Therefore, before the surgical interventions for implant placement, each case should be carefully evaluated taking into account specific patient characteristics [3,6,19].

By giving priority to simpler and less invasive procedures, short implants can be considered as an alternative therapy in cases of atrophic alveolar ridges as they may entail fewer interventions, lower patient morbidity, reduced costs and a shorter time of treatment [3,4,5,10,20]. Moreover, the improvement and better understanding of surgical techniques, implants’ macro and microgeometry, prosthesis design and biomechanics support the use of short implants [8].

In the past years, manufacturers have improved implants’ macroscopic topographies and surface characteristics. While the first studies used turned surfaces, more recent studies have reported data on rougher surfaces (such as sandblasted and acid-etched (SLA)) aimed to maintain the extent of the bone-to-implant contact, thus mitigating the effects of a reduced implant length [2,9]. One of the major concerns about short implants was the greater crown-to-implant ratio, which could jeopardize implant survival due to marginal bone loss when non-axial forces are applied [8,21]. However, some authors concluded that this is not a predictor factor of marginal bone loss or implant survival [22,23,24,25]. A recent systematic review reported that a crown-to-implant ratio between 0.86 to 2.14 of single-tooth, non-splinted, implants did not present higher risk of biological or technical complications [22]. A greater stress concentration occurs at the crestal level instead of at the apical level of the implant. It is described that the use of wider implants is more effective than the use longer implants in reducing the forces at the bone–implant interface thus leading to less crestal marginal bone loss in the long term [26]. Moreover, some authors reported that narrow implants (3.3 mm) may be more susceptible to fracture due to fatigue than wider implants are, mainly in the posterior mandible [27,28]. The prosthodontic cantilevers may also represent a biomechanical challenge. The use of cantilevers in posterior regions when the rehabilitation of short implants is performed should be carefully managed, since these may compromise implant stability by increasing marginal bone loss [24].

The definition of “short” implant varies between authors, ranging from less than 10 mm to 8 mm, 7 mm or 6 mm in length [3,4,9,10,17,29,30,31,32,33,34]. However, Renouard and Nisand [30] advocated that its definition should consider the intraosseous length of the implant, since it can be placed at different horizontal levels and that, thus, a short implant should be defined as an implant with an intraosseous length of ≤8 mm.

Up to this date, long-term data about the predictability of short implants based on large sample sizes are scarce. Therefore, this single-center retrospective study aimed to evaluate the survival rate of short SLA-surfaced implants, namely 8 mm length bone-level implants and 6 and 8 mm length tissue-level implants, with at least 120 days of follow-up. The tested null-hypothesis was that there is no difference in the survival rate of short bone-level and tissue-level implants.

## 2. Experimental Section

### 2.1. Study Design and Patient Selection

The present retrospective study followed the protocols of the World Medical Association Declaration of Helsinki (2013 version), was approved by the Ethics Committee of the Lisbon Implantology Institute (II2018-02 from February 2018) and was prepared according to the Strengthening the Reporting of Observational Studies in Epidemiology (STROBE) guidelines [35].

The sample of this study was obtained from the clinical records database of a single private practice in Lisbon, Portugal with all the included patients signing an informed consent form for clinical data collection. The database was reviewed, and the study was conducted within the quality control guidelines of the institution, with all data collected according to strict guidelines, researchers certified for good practical guidelines according to ICH GCP and the interventions performed by three experienced surgeons.

The following inclusion criteria were applied: (1) be at least 18 years of age; (2) have at least one edentulous space requiring implant placement; (3) have indication to use implants of 8 mm or less in length; and (4) have good systemic health (ASA score ≤II) [36]. Because this was a pragmatic study performed in a private clinical setting, patients with active smoking habits, evidence of parafunctional habits (i.e., bruxism) and/or sub-optimal oral hygiene were not excluded. According to the practice guidelines, each patient was thoroughly informed about the surgical and prosthodontic procedures and signed an informed consent agreement before the surgical intervention.

### 2.2. Surgical Procedures

The case selection criteria included patients treated with short dental implants who had been followed up for at least 120 days since the surgery and who complied with mandatory follow-ups of at least one visit per year. There were no restrictions regarding the anatomical location of the implants in the maxillae. All patients had a routine oral hygiene appointment before implant placement and every four months after the surgery.

In order to set the adequate treatment plan, bone availability and quality were evaluated in all cases before surgery with a diagnostic cone-beam computed tomography (CBCT) (Planmeca ProMax, Planmeca, Helsinki, Finland) with a large field of view (FOV) and a 0.20 mm voxel size, at 80 kV and 15 mA, within an exposure time of 12 s.

All patients included in the study were treated by three experienced surgeons with Straumann (Straumann, Basel, Switzerland) tissue-level implants of 6 (TL6) or 8 mm (TL8) or Straumann bone-level tapered implants of 8 mm (BLT8), using the manufacturer’s standardized surgical procedure. Surgeries were performed between January 2017 and July 2019. After local anesthesia with articaine hydrochloride with epinephrine (1:200,000), a full-thickness flap was reflected, and the implant sites were marked with the initial drill. Then, the implant site was further prepared following the manufacturer’s instructions. When a contour augmentation was necessary, minor regenerative procedures were performed with bone substitute material and a collagen membrane (the same substitute material and collagen membrane at all study sites). The wound was closed with resorbable sutures (Vicryl 4-0, Johnson & Johnson, Brunswick, NJ, USA). The implants were placed using a two-stage submerged surgical protocol or a one-stage non-submerged surgical protocol, at the surgeon’s discretion. The time of loading was based both on primary stability (insertion torque ≥32 Ncm) and the clinical decision of the surgeon (based on aesthetic demand, location, type of rehabilitation and function). In cases of delayed loading, the healing phase corresponded to a minimum of 3 months.

According to the practice guidelines, the follow-up visits were scheduled at one week and one, three and six months after the intervention, and then once a year. In each follow-up visit, the biological and technical complications were assessed, and panoramic radiographs were taken when necessary.

### 2.3. Variables

Data concerning patient characteristics (gender and age), periodontal status (healthy, supportive periodontal treatment before or during the follow-up period), date of implant placement, implant characteristics (type, length, width), implant placement area, type of edentulism (single, partial, total), additional regeneration procedures, time of loading, failure (implant and prostheses) and final follow-up appointment were retrieved from the clinical records. Periodontal assessment was performed with a periodontal probe (Hu-Friedy^®^ Inc., Chicago, IL, USA) to collect the probing pocket depth and bleeding on probing (present or absent). Patients with active clinical periodontal disease, expressed by probing pocket depths ≥5 mm and bleeding on probing, were referred to a specialist in periodontology. Patients with probing depths <5 mm and absence of bleeding on probing were considered healthy [37,38].

The predictor variables to be tested were the type of implant (tissue level or bone level), the implant length (6 or 8 mm) and implant width (4.1 or 3.3 mm) and. The primary outcome was the implant and prostheses survival.

All the patients were followed-up clinically and radiographically to identify any signs of implant failure. The implant was considered as a failure if it had been lost from any cause, either biological (failure to achieve osseointegration or loss of acquired osseointegration) or biomechanical, or if there was persistent pain, mobility or an untreatable infection, being classified as follows: (1) early failure, need to be removed before achieving six or three months in function for implants placed with and without regenerative procedures, respectively; (2) late failure, if removed after prosthetic loading and following a successful osteointegration period [39]. Thus, any implant that presented evidence of peri-implant radiolucency, clinical mobility, persistent pain, untreatable infection or increased probing depth was considered as an implant failure.

### 2.4. Data Analysis

Data and statistical analysis were conducted using the software IBM SPSS Statistics V24 for Mac (SPSS Inc. Released 2016. SPSS for Mac, Version 25.0. Chicago, SPSS Inc.).

Descriptive statistics were performed for patient demographics, implant characteristics (length, width), periodontal status, implant placement area and type of edentulism (single, partial, total). Absolute and relative frequency distributions were calculated for qualitative variables, and mean values and standard deviations for quantitative variables.

The implant was considered as the unit of analysis. A Cox regression model was conducted, adjusted for a time-dependent covariate and the outcome factors (implant type, length and width). The cumulative survival rate was assessed by time-to-event analyses (Kaplan–Meier estimator). The methodology and statistical analysis were reviewed and performed by an independent statistician.

## 3. Results

A total of 513 patients (329 women, 184 men) with a mean age of 58.00 ± 12.44 years at the time of surgery (min = 23; max = 90; range = 67) were eligible for this study (Table 1).

**Figure 1:**
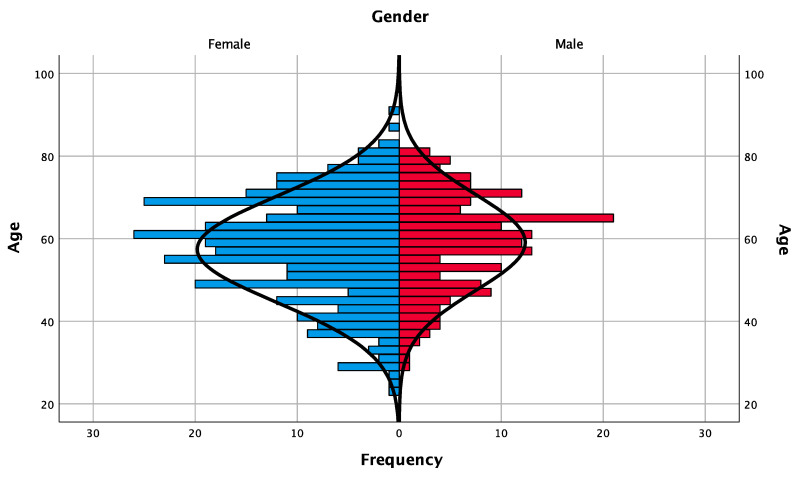
Demographic pyramid of the frequency distribution of patients according to gender and age.

A total of 1008 implants were installed. Figure 1 and Table 1 show frequencies regarding the patients’ characteristics (gender, age and periodontal status). Implant characteristics (diameter, type of rehabilitation, location, time of loading and failure) are depicted in Table 2. The mean follow-up time of the dental implants was 21.57 ± 10.77 months after placement. Regarding the periodontal status, 87.9% of the patients were considered healthy, while 12.1% received supportive periodontal treatment previously to the surgery and/or during the follow-up period.

A total of 476 implants were placed in the BLT8 group (mean follow-up time of 18.43 ± 9.02 months), 260 in the TL6 group (mean follow-up time of 25.09 ± 11.51 months) and 272 in the TL8 group (mean follow-up time of 22.51 ± 12.29 months). The most frequent implant width was 4.1 mm (78.17%). The posterior maxilla and the posterior mandible were the most frequent locations for short implants, with 318 and 544 implants, respectively. Of all implants, 31.94% were single unit, while 44.15% and 23.91% were integrated into partial and total rehabilitations, respectively. Only in 13.5% of the cases, a minor regeneration procedure was necessary. The implants with 3.3 mm width were predominantly placed for partial and total rehabilitations, 45.3% and 36.9%, respectively. Moreover, 42.3% of these implants were located in posterior mandible and 21.8% in posterior maxilla.

During the evaluation period, 14 implants were lost within the healing period after surgery (early loss due to failure to achieve osteointegration) and 8 after that period (late loss: 5 due to mobility, 2 due to continuous pain and 1 due to infection), in 22 periodontally healthy patients. Most of the failed implants were located in the posterior maxilla or mandible (*n* = 8 and *n* = 9, respectively).

A Cox regression was performed for implant type, length and width. Type, length and width were not significant for the Cox regression model (*p* = 0.404; *p* = 0.478 and *p* = 0.695, respectively). The obtained plots of survival functions show that the model is adequate, since the distance between lines keep constant.

**Figure 2:**
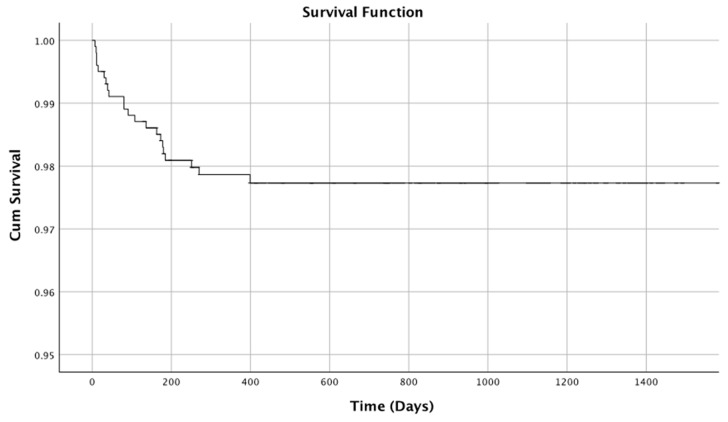
Cumulative implant survival rate by time-to-event analyses (Kaplan–Meier estimator).

Therefore, neither type, length nor width significantly influence the implant survival, and thus, the survival rate can be calculated for all implants included in the sample. The cumulative implant survival rate was 97.7% ± 0.5% (Figure 2).

Regarding prostheses survival, 693 prostheses were evaluated with eight implants lost during follow-up (late loss), i.e., in 62.5% of the cases, the prosthesis could be maintained corresponding to an overall prosthesis survival rate of 99.57%.

## 4. Discussion

During the past years, manufacturers have developed modifications in implant surface treatment techniques and implant designs that have a positive impact on the prognosis of short dental implants [2,9]. Short implants were initially defined as implants smaller than 10 mm in length [9,33]. However, this first definition has been questioned, and different new proposals have been described—implants shorter than 8, 7 or even 6 mm [17,29,34,40]. According to Renouard and Nisand [30], it is not the length of the implant that should be considered but rather their intraosseous length, since the implant can be placed at different horizontal levels; and, therefore, a short implant should be defined as an implant with an intraosseous length of 8 mm or less.

The success of short dental implants remains a controversial topic, mainly due to the variability in the literature regarding not only their definition but also the study protocols used. Recent systematic reviews and meta-analyses have highlighted the predictability of short dental implants with survival rates comparable to longer implants—or the so-called “conventional implants”—when placed in pristine or augmented bone [3,4,9,10,40,41,42,43]. On the other hand, some clinical studies have reported a higher risk of failure for short implants when compared with longer implants [17,44].

Therefore, this retrospective study aimed to evaluate the survival rate of short SLA-surfaced dental implants using the anatomical location, type or length of the implants as predictor variables. The study was conducted in a private clinical center under strict quality-control protocols and had a pragmatical design in a real-world setting, thus increasing its external validity [45,46,47].

To the best of our knowledge, this is the largest single-center retrospective study on the survival rate of short dental implants, thus overcoming inter-center variability and the sample size limitation of previous studies [5,9,10,20,40].

The results of this study reported a high two-year cumulative survival rate (about 97.70%), without statistical differences between groups and outcomes. This rate is in agreement with similar studies [2,20,32]. A long-term study [32] that evaluated 8 and 6 mm length tissue-level implants reported a 5 and 10 year cumulative survival rate of 98.7% and 98.3%, respectively, which is in accordance with the two year cumulative survival rate range obtained in this study. A multicenter retrospective study with 6 mm SLA-surfaced implants reported higher predictability when placed in the mandible and splinted, with a 96.4% 6 year cumulative survival rate [2]. More recently, a long-term follow-up study (15 years) reported a cumulative survival rate of 93.3% [20]. According to several authors, these high survival rates could be due to implant surface modifications that guarantee higher bone-to-implant contact, with rough surfaces having a larger contact area than smoother ones at the microscopic level [9,32], thus increasing the area available for osteointegration, which could compensate the potential adverse side effects of a reduced implant length. In our study, micro-rough SLA-surfaced implants were used, which may partially justify the high cumulative survival rate observed since this surface treatment had already yielded favorable results, both in clinical and experimental studies [48]. In fact, the surface treatment can directly influence the performance of short implants, as reported by Weng et al. in a prospective multicenter clinical trial with machined-surfaced implants, in which the authors reported an implant failure rate of 8.8% with <10 mm in length, at the 6 year follow-up [49].

According to the literature, several factors may influence the risk of implant failure, namely, bone quantity and quality, implant location (maxilla vs. mandible), implant design (micro and macroscopic design), type of rehabilitation (single units vs. multiple units; splinted vs. non-splinted), occlusal loading and surgical factors (such as osteotomy preparation) [2]. However, in this study, when the Cox regression was adjusted for the variables implant type, length and width, the authors did not detect any statistically significant influence between groups and outcomes.

**Figure 3:**
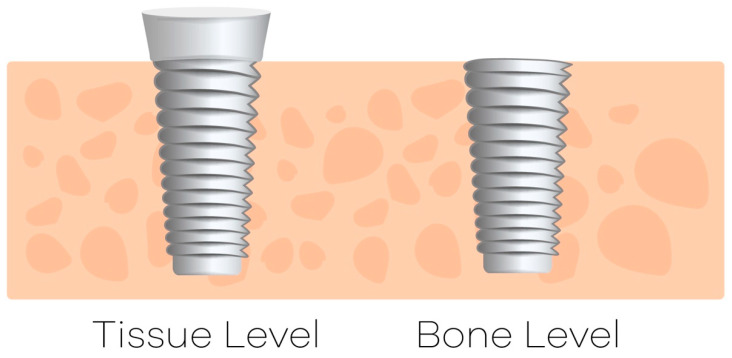
Location of the implant–abutment junction in relation to bone crest for tissue- and bone-level implants.

Our study intended to compare not only different lengths (6 and 8 mm) but also different types of two-piece implants: the bone-level implant, installed sub- or equicrestal, and the tissue-level implant, installed at the soft-tissue level (Figure 3). Although both implants present the same surface treatment, they differ in macrogeometry, surgical protocol and location of the implant–abutment junction in relation to the bone crest and soft tissue. Hermann et al. found that the existence and the level of this microgap can affect the bone level around the implant. While the submerged, two-piece implant approach presented crestal bone changes dependent on the location of the microgap, minimal or no changes were observed in crestal bone level of non-submerged, one-piece implants [50]. Microbial colonization of the microgap, micromovements of the abutment or an interruption in blood supply when abutments are placed has been suggested as possible mechanisms for crestal bone changes [50]. Piattelli et al. confirmed that when the microgap is placed coronally from the alveolar crest less bone resorption occur [51]. Thus, implants with a transmucosal profile (the tissue-level implant) present limited bone loss, which in the long term could be associated with different survival rates.

Similarly to other studies, our results demonstrated that implant diameter was not a statistical significant factor for implant survival [25]. The Straumann 3.3 mm diameter implants used in our study were the first implants manufactured with titanium–zirconium alloy (Ti-15Zr, Roxolid, Straumann AG, Basel, Switzerland), with studies demonstrating that it could present 40% better resistance to fatigue stress than titanium [52].

Although the results of our study presented high survival rates at the two-year follow-up for the different types and lengths of the evaluated implants, caution should be taken when assessing the obtained results since failure rates could depend on time in function. A recently published meta-analysis [53] evaluated the short implants’ (≤6 mm) failure rates based on time in function, and the results demonstrated a time-dependent decrease in the survival rate of single short implants in the posterior area. Furthermore, some long-term studies reported that, between the third and fifth years in function, the risk of short implant failure increases significantly [33,44]. Additionally, the fatigue stress failure may occur after a long period of continuous thermal and loading cycles. These data reinforce the importance of studies with longer follow-ups.

The authors were well aware of the limitations of this study due to its retrospective nature and the fact that the marginal bone loss was not assessed. Therefore, they focused on obtaining a large enough sample size with a patient-centered relevant outcome, which, in this case, was the implant survival.

## 5. Conclusions

According to the data reported, the evaluated SLA implants demonstrated high survival rates during the follow-up period considered, without statistical differences between the types, lengths and width studied. The obtained results are comparable to those of standard-length implants reported in the literature. Further studies with longer follow-ups are required to evaluate the survival rates of short dental implants in the long term.

## Figures and Tables

**Table 1:** Patient’s characteristics in each implant group (*n* = patient).

	Implant Type		Bone Level (BL)	Tissue Level (TL)	
	Implant Length		8 mm	6 mm	8 mm
Patient-based analysis	Gender	Male	88	53	68
		Female	159	115	111
	Age	Mean	57.65 ± 12.31	58.32 ± 12.59	58.25 ± 12.57
	Periodontal Status	Healthy	213	140	148
		Supportive periodontal treatment before surgery	25	18	23
		Supportive periodontal treatment during follow-up period	9	10	8

**Table 2:** Implant-based analysis in each implant group (*n* = implant).

	Implant Type		Bone Level (BL)	Tissue Level (TL)		Total
	Implant Length		8 mm	6 mm	8 mm	
Implant-based analysis	Implant Width	3.3 mm	184	-	36	220
		4.1 mm	292	260	236	788
	Type of rehabilitation	Single	127	78	117	322
		Partial	163	145	137	445
		Total	186	37	18	241
	Location	Anterior Maxilla	85	2	9	96
		Posterior Maxilla	155	89	74	318
		Anterior Mandible	39	2	9	50
		Posterior Mandible	197	167	180	544
	Time of loading	Immediate	123	8	8	139
		Delayed	353	252	264	869
	Implant Failure	No	464	254	268	986
		Yes	12	6	4	22
